# A Steak-Pie Story

**Published:** 1889-07-13

**Authors:** 


### A STEAK-PIE STORY.

The wife of Dr. X. had a pie made of steak and pigeons at the beginning of the present hot weather. It was partaken of on the day of cooking without harm to anyone. On the second day, when it was eaten cold, three persons suffered severely from diarrhœa, for which they could discover no cause except the pie. In a few days the incident was forgotten. A month later another pigeon-pie with steak was made. Again on the day of cooking no harm resulted. On the second day Dr. X. noticed that a portion of the pigeon looked peculiar, though it had no tainted smell, and had only been killed the day before. He, however, remarked at the time that he hoped there would be no confirmation of the former suspicion that the steak-pie had given rise to diarrhœa. But the hope was vain. Within a few hours three persons, as before, were again attacked, and much more seriously, one of them, indeed, alarmingly. A fourth who partook escaped, as she had done on the former occasion. Thereupon both Dr. X. and his wife resolved to have no more steak-and-pigeon pie so long as the hot weather continued, unless it were a small one which could be eaten at a single sitting. This strictly moderate conclusion, we think, was amply justified by the facts. In very hot weather ordinary persons, who have not got an icehouse in which to keep food, should, as a rule, buy no more meat and cook no more than is sufficient for the needs of the day.

